# *Journal of Cheminformatics*, ORCID, and GitHub

**DOI:** 10.1186/s13321-019-0365-4

**Published:** 2019-07-08

**Authors:** Egon Willighagen, Nina Jeliazkova, Rajarshi Guha

**Affiliations:** 1 Department of Bioinformatics – BiGCaT, NUTRIM, Maastricht University, P.O. Box 616, UNS 50 Box 19, 6200 MD Maastricht, The Netherlands; 2 Ideaconsult Ltd, Sofia, 1000 Bulgaria; 3 Vertex Pharmaceuticals, 50 Northern Avenue, Boston, MA 02210 USA

Two years ago, Rajarshi Guha and Egon Willighagen took over the Editor-in-Chief roles for the *Journal of Cheminformatics* from Christoph Steinbeck and David Wild [1]. This year our journal reached the age of 10: on 17 March 2009, Wild welcomed us to a new journal for cheminformatics [2], a 100% open access journal, which was still very new for chemistry at the time. In those 10 years, the journal published 939 papers, of which 286 are research papers and 110 are software papers. To help us with the handling of all submissions, we were joined by Nina Jeliazkova as our first Associate Editor in 2018.

When Guha and Willighagen took over, they set out to further the field in its transition to Open Science [1]. The first thing we did was to start a Twitter account, where we have since been tweeting the articles, as an alternative to the RSS feed of new articles. The Twitter account currently has more than 700 followers. The second thing we did was start encouraging more authors to provide their ORCID identifiers [3]. We started requesting the corresponding author to provide their ORCID identifier and we updated our Editorial Board page with ORCID identifiers for our board members (https://jcheminf.biomedcentral.com/about/editorial-board).

And starting this year, we extend this layer of FAIR-ness of our journal by requiring ORCID identifier for *all* authors in a paper [4]. The manuscript submission system does not allow us to ask for all ORCIDs on submission, and so currently they need to be provided in the manuscript itself. The adoption has a lot of benefit [5, 6] and a particularly important benefit for the *Journal of Cheminformatics* is that it allows us to follow young researchers that may be potential reviewers. Of course, the same applies to our readers who can now much more easily find other research by the authors of an article they are interested in, e.g. with EuropePMC [7] and Scholia [8].

**Fig. 1:**
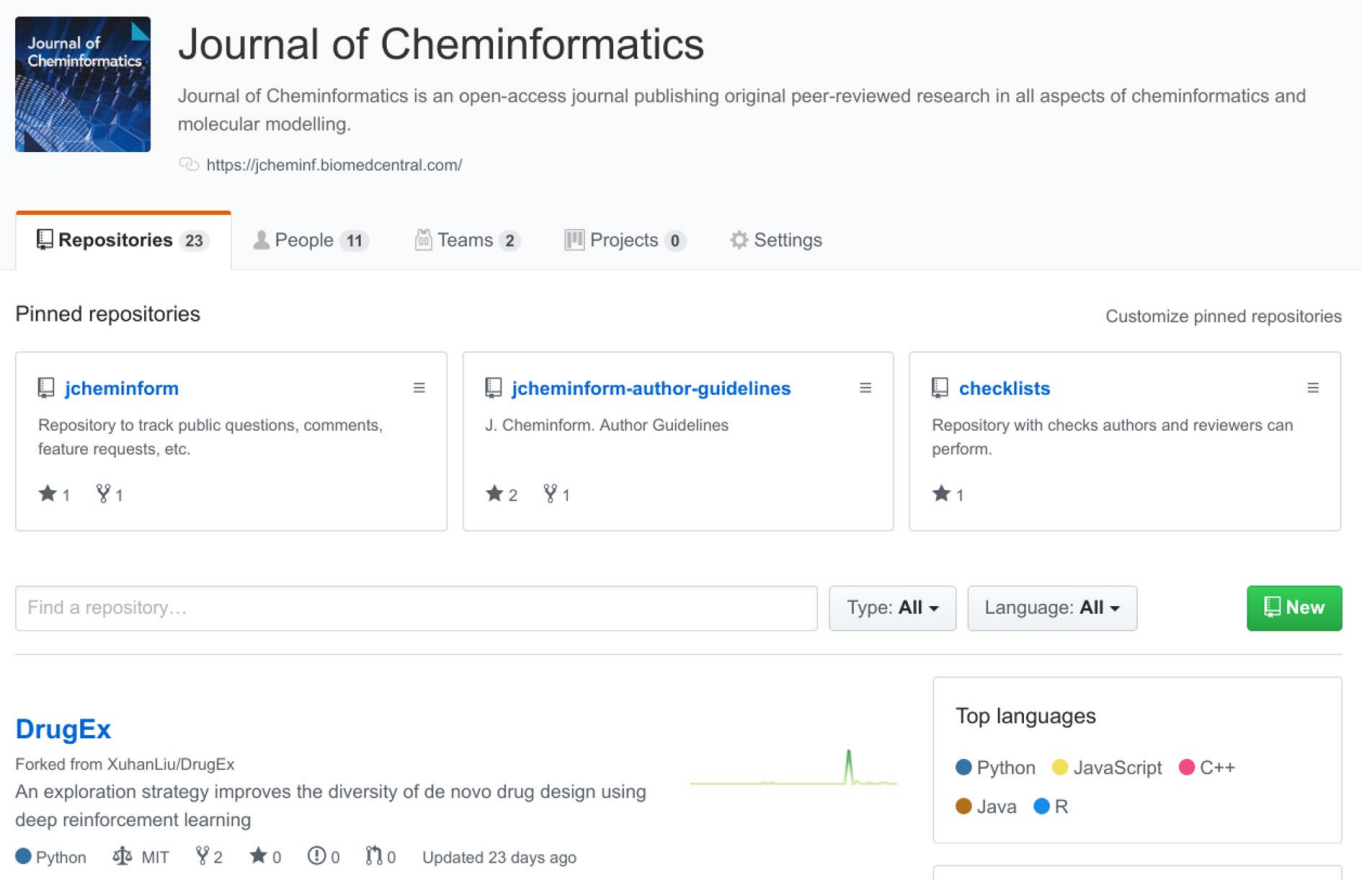
GitHub page for the *Journal of Cheminformatics*, showing various repositories about the journal, and the DrugEx repository, one of the git repositories associated with a recent article by Liu et al. [11]

A fourth change we recently made was following the example set by *eLife* [9], and we started a GitHub Organization for the journal: https://github.com/jcheminform (see Fig. 1). Following the concepts of Open Science, we here share our documentation like our journal-specific Author Guidelines (https://github.com/jcheminform/jcheminform-author-guidelines) and have and Issue Tracker to allow authors and readers to give feedback (https://github.com/jcheminform/jcheminform/issues). And we here fork source code repositories associated with *Journal of Cheminformatics* articles. This project is still under development, and we have yet to iron out how we want to use this. Feedback is most welcome in our new Issue tracker. Smiling Emoji.

Finally, we like to stress our continued gratitude to our Editorial Board, all our reviewers, and all our authors. Without them, the *Journal of Cheminformatics* would not be a viable journal. It is with your support that we can continue promoting and supporting Open Science in chemistry. We also thank and say goodbye to Samuel Winthrop, who has been publishing editor for several years but now stepped down. We acknowledge we have a long way to go, which becomes crystal clear when you reread Steven Bachrach’s vision from 2009 [10].
